# Human *O-*GlcNAcase catalytic-stalk dimer anchors flexible histone binding domains

**DOI:** 10.21203/rs.3.rs-6197257/v1

**Published:** 2025-04-01

**Authors:** Sarah B. Nyenhuis, Agata Steenackers, Jenny E. Hinshaw, John A. Hanover

**Affiliations:** 1 Laboratory of Molecular Biology, NIDDK, National Institutes of Health Bethesda MD 20851; 2 Laboratory of Cell and Molecular Biology, NIDDK, National Institutes of Health Bethesda MD 20851; 3 These authors contributed equally to the manuscript

## Abstract

*O-*GlcNAc transferase (OGT) and *O-*GlcNAcase (OGA) perform essential functions in signaling, epigenetics, and transcription. Although thousands of proteins are specifically *O-*GlcNAc modified, the molecular features recognized by the enzymes of *O-*GlcNAc cycling remain poorly defined. Here we solved the structure of the long isoform of human OGA by cryo-electron microscopy providing a physiologically relevant platform to study the enzyme. The 3.63 Å structure of the dimeric catalytic-stalk dimer differs notably from existing crystal structures. Intriguingly, a low-resolution structure of the OGA-L exhibit densities corresponding to the C-terminal pseudo-HAT domains suggesting substantial flexion of these domains relative to the catalytic-stalk dimer. To explore the role of these domains we found that OGA-L binds to a small subset of the 384 modified histone tails on a commercial array. High specificity binding was observed with modified histone H3K36 peptides, and H4 acetylated peptides. Based on these findings, we propose OGA-L is poised to bind two modified histone tails of nucleosomes in open chromatin but excluded from repressive chromatin. Increased local concentration and activation of OGA-L coupled with its intrinsic conformational flexibility facilitates the removal of *O-*GlcNAc from target proteins in proximity such as the intrinsically disordered CTD domain of RNA Polymerase II. This model is consistent with OGA-L acting as a ‘reader’ of histone modifications linked to development, transcriptional activation, transposon silencing, and DNA damage repair.

## Introduction

The *O-*GlcNAc transferase (OGT) enzyme catalyzes the addition of *O-*GlcNAc to target proteins, while *O-*GlcNAcase (OGA) removes this modification. These two enzymes have become important subjects of research following the discovery of *O-*GlcNAc modification and its crucial role as a post-translational modification (PTM) in regulating protein activity, localization, and degradation ^1-5^. Furthermore, *O-*GlcNAcylation is integral to many cellular signaling pathways, and its dysregulation has been linked to conditions such as insulin resistance, diabetes, cancer, lupus, and neurodegenerative diseases ^4,6-10^. In humans, mutations in OGT are associated with X-linked intellectual disability with alteration is OGA levels observed in some of these patients ^5,11-15^. *O-*GlcNAcylation targets serine and threonine residues on thousands of intracellular proteins and shows considerable crosstalk with kinase cascades and phosphorylation ^3,9,16^. Unlike phosphorylation, which is regulated by numerous kinases and phosphatases, *O-*GlcNAcylation is mediated by only two enzymes: OGT and OGA. Despite the well-documented importance of OGT and OGA, the molecular mechanisms underlying substrate recognition by these enzymes remain poorly understood. A significant challenge has been the absence of structural information, particularly for the full-length forms of these proteins. Human OGA (hOGA) is located on chromosome 10, with two major isoforms: OGA-L (long), a 103 kDa nucleocytoplasmic protein, and OGA-S (short), a 75 kDa nuclear protein associated with nascent lipid droplets ^4,17^. The N-terminal domain of OGA forms an α/β barrel with hydrolase activity, while the stalk domain, composed of α-helices, contributes to OGA dimerization, as revealed in recent structural studies ^18-20^. The longer isoform differs from the short isoform in containing a “pseudo-HAT” domain with a predicted fold like MYST domain histone acetyltransferases ^21^. This domain of the protein lacks the ability to bind Acetyl-CoA or catalyze the acetyl transfer reaction. The C-terminal pseudo-histone acetyltransferase domain remains structurally uncharacterized in mammalian *O-*GlcNAcase ^21^. Notably, the constructs used in the latest structural studies included the catalytic domain and part of the stalk domain, with some regions replaced by linkers but lacked the C-terminal HAT domain. To date, the full-length structure of OGA is unavailable, and the role of the HAT domain in substrate recognition and enzyme function is unclear.

In addition to key roles in cytoplasmic signaling, *O-*GlcNAc cycling is an important contributor to epigenetic programming and the regulation of gene expression ^2,5,22-28^. In mammals both OGT and OGA are essential for normal development ^24,29^. Both OGT and OGA are involved in processes linked to post-translational alterations in histones and higher order chromatin structures such as polycomb repression, transcriptional activation and DNA damage repair ^24,30-35^. The direct *O-*GlcNAc modification of histones themselves has been somewhat controversial ^36,37^, but the interconnections between the histone code and *O-*GlcNAcylation are clear from genetic and biochemical studies ^2,30,38,39^. Notably, OGT is associated with the polycomb complex PRC2 governing H3K27 methylation ^24,30-33,38^. OGT is required for polycomb repression in the well-studied *Drosophila* system ^32,33^. In this system, loss of *O-*GlcNAcase leads to a spread of polycomb repression at new sites in the genome ^30^ and transcriptional deregulation ^22,30^. In addition, OGA plays a role in transcriptional activation associated with activating histone modifications such as H3K36 methylation and acetylation ^22,27,30,40,41^. Despite the well-documented importance of OGT and OGA in regulating gene expression, the molecular mechanisms underlying substrate recognition by these enzymes remain poorly understood. Understanding how the structure of the enzymes of *O-*GlcNAc cycling contribute to these biological functions is an important challenge. The gene encoding OGT in humans is on the X chromosome and is subject to X-inactivation in females making its role in transcriptional repression particularly interesting ^24,42-44^. The structure of the OGT protein has been studied extensively by crystallography and cryo-EM leading to a model of catalysis, dimerization and substrate recognition ^45-50^. In addition, one of these recent studies suggested how OGT and OGA may interact structurally to produce an autoinhibitory complex ^45^.

Here we solved the cryo-EM structure of full-length human *O-*GlcNAcase to a resolution of 3.63 Å, and locally refined OGA A-chain to 2.98 Å and OGA B-chain to 3.05 Å. The structure reveals a dimer interface that differs from the existing crystal structures of the dimeric catalytic-stalk dimer - but retains the features of the catalytic core. The C-terminal portion of the molecule containing the pseudo-HAT domain is preceded by an intrinsically disordered region which confers conformational flexibility bridging the two domains. The structure reveals that the C-terminal HAT domain adopts multiple conformations. A lower resolution structure of the OGA-L exposed the most common configurations of the two HAT-like domains. To gain insight into function of domains of the *O-*GlcNAcase, we performed biochemical, enzymatic, and structural studies on the individual domains and the intact OGA-L. Both short and long isoforms of *O-*GlcNAcase show enzymatic activity towards model substrates. Histone arrays experiments, interrogating 384 combinations of modified histone peptides using the intact molecule and individual domains, suggest that the HAT domain and full-length *O-*GlcNAcase specifically bind to a limited subset of histone tails. The most specific binding occurred with histone H3K36 peptides and their di- and trimethylated forms.

Based on these structural findings, we propose that *O-*GlcNAcase may stably recognize peptide targets including histone tails in nucleosomes with its HAT domain, while the intrinsically disordered linker region allows flexibility for the catalytic-stalk dimer to recognize and deglycosylate adjacent targets up to ~ 100Å.

## Results

### Cryo-EM structure of Full-length Human *O-*GlcNAcase (OGA-L)

**Fig. 1:**
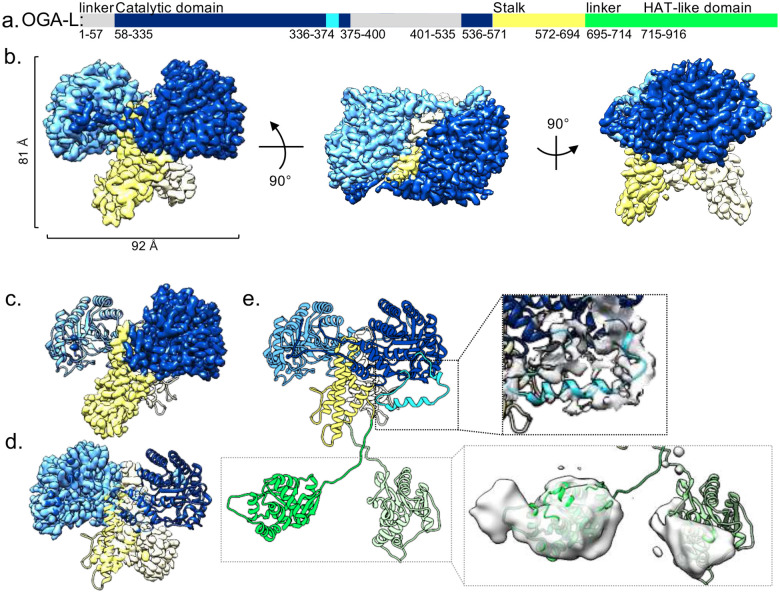
cryo-EM structure of long *O-*GlcNAcase (OGA-L) isoform. **a**, Sequence diagram of OGA-L colored by domain, highlighting flexible regions. OGA catalytic domain: dark blue, unstructured regions: gray, flexible helix: cyan; stalk: yellow; linker and HAT-like domain: green. **b,** cryo-EM map of OGA catalytic-stalk dimer and stalk domains colored as in **a** with dimensions of the dimer highlighted. **c,d** cryo-EM maps of locally refined OGA A-chain (**c**) and B-chain (**d**) with catalytic and stalk domains colored as in (**b**). **e,** A model of the resolved and partially resolved regions of OGA-L (left) and the density of regions which are resolved to low resolution are shown (light gray) (right). The flexible helix in the catalytic domain is highlighted in cyan and a model of the flexible HAT-like domains (green) is shown fitted into low-resolution density.

Here we present a 3.63 Å cryo-EM structure of full length human *O-*GlcNAcase (OGA-L) in the apo state exhibiting the canonical interlocking dimer interface comprising the catalytic (blue) and stalk (yellow) domains (Fig. 1a,b, Supplementary Fig. 1). The resolution was further improved with local refinement focused on the monomers ^51,52^ to 2.98 Å for OGA A-chain (Fig. 1c, dark blue) and 3.05 Å for OGA B-chain (Fig. 1d, light blue). This allowed us to build an atomic model of the catalytic-stalk dimer (Fig. 1c,d). A representative fit of the model to the map, highlighting residues 113-124 in the catalytic domain, map is shown in Supplementary Fig. 1c and the interfacial residues in the dimer interface are highlighted as sticks with hydrogen bonding residues (orange) in Supplementary Fig. 1d and Supplementary Table 1. The buried surface area of the dimer interface is 4203.3 Å^2 53^. The catalytic-stalk dimer exhibits a minor asymmetry with a displaced helix in the A-chain (Fig. 1e, cyan). Comparison between our OGA-L cryo-EM structure with existing truncated crystal structures suggest that inclusion of the flexible regions within the catalytic-stalk dimer and C-terminal HAT region generate notable differences in the observed dimer interface (Supplementary Fig. 2, Supplementary Video 1). Removal of these flexible regions likely explains the difference observed in the dimer interface. As may be expected, AlphaFold2 structural predictions of the full-length OGA-L better agree with our full-length OGA-L cryo-EM structure than the truncated OGA crystal structures.

Comparing the catalytic active site between our cryo-EM model of full-length OGA-L (blue) with eleven crystal structures (white) and one previous cryo-EM structure revealed only small differences (Supplementary Fig. 2a-n). This is apparent with both apo and ligand bound crystal structures. The existing apo crystal structures (5m7r, 5vvo, 5uhk) showed little deviation or evidence of side-chain rearrangement compared to the cryo-EM map (Supplementary Fig. 2a-c, m). We also compared our structure to crystal structures with the inhibitors ThiametG bound (5m7s,5un9, 5uhl), and PugNAc bound (5m7t, 5uho) as well as the S-linked CKII peptide target (8p01) (Supplementary Fig. 2d-i, n). The relative position of sidechains in the active site also showed little movement or rearrangement. In addition, we compared the cryo-EM map to crystal structures incorporating the inhibitors Ceperognastat (9ba8), and Aminothazole-1 (9ba9) (Supplementary Fig. 2j,k). Here, there was substantial variation of the overall dimeric structure, but little deviation in the active site. Finally, a recent cryo-EM structure of the OGA monomer in complex with OGT (7yeh) also fit well into our cryo-EM structure with sidechains in the resolved active site positioned similarly in both structures. In each case, the sidechains visible in our cryo-EM map of the active site conform closely with the positions of residues in the active site of all crystal structures (Supplementary Fig. 2a-k, n,m) and the one cryo-EM model (Supplementary Fig. 2l).

**Fig. 2:**
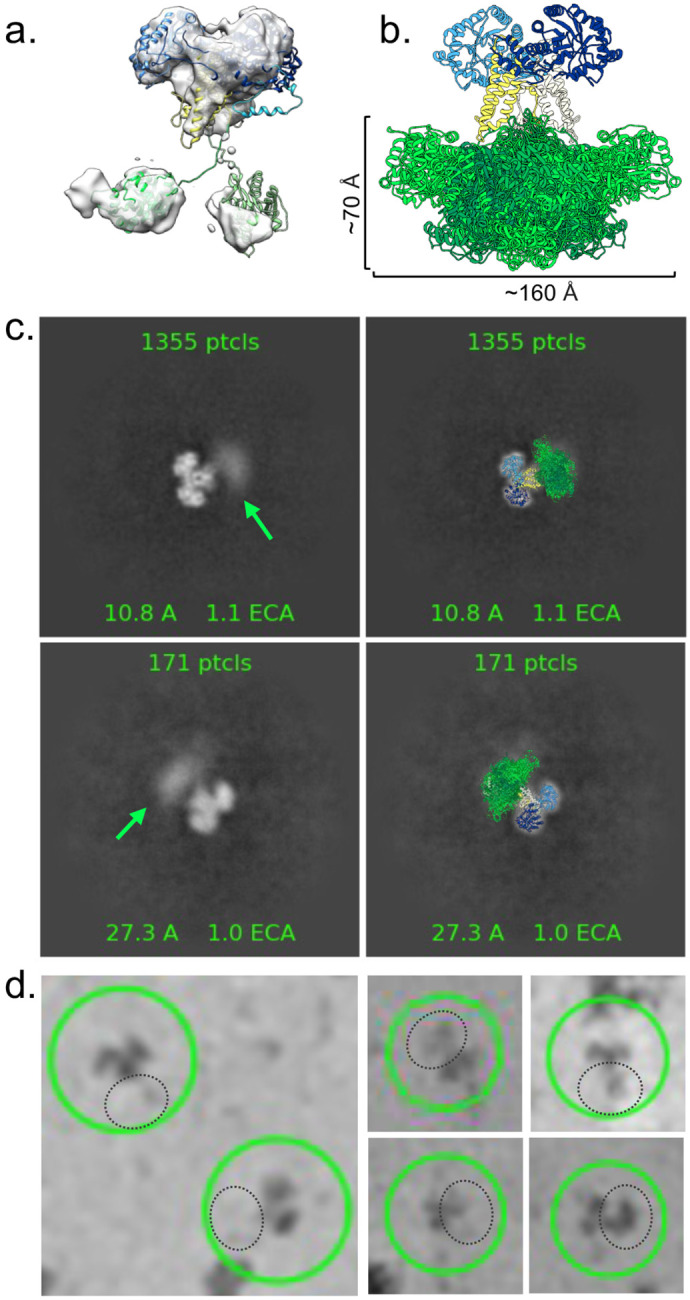
Flexibility of the HAT-like domain. **a,** A low-resolution cryo-EM map reveals density in the region of the HAT-like domain. A representative model, colored by domain: catalytic domain, dark blue; stalk, yellow; linker and HAT-like domain: green shades, is fit into the map. **b**, Model colored as in (a). An ensemble of HAT-like domains produced by the FastFloppyTail application in Rosetta^53^. Dimensions were measured for the ensemble and 2D classes produced in cryoSPARC^50,51^. **c,** Left, 2D class averages of OGA exhibit clouds of density, below the catalytic-stalk dimer, that are consistent with flexible HAT-like domains (green arrow). Right, representative models of OGA colored as in (**a**) overlaid with the 2D class averages. **d,** Single particles of OGA showing different positions of the HAT-like domains produced from manual selection in cryoSPARC. OGA particles are circled (green) with potential HAT-like domains indicated by dashed black circles.

In our high-resolution OGA structure, there is a lack of density associated with the C-terminal domains of the protein. However, extensive 3D classification yielded a low-resolution map with additional density that likely represents the C-terminal HAT-like domains of the OGA-L dimer (Fig. 1e, lower right, Fig. 2a). In addition, expanded 2D classification of OGA, with fewer particles in each class, also revealed clouds of density below the catalytic-stalk dimer (Fig. 2c, green arrows) and in individual particles (Fig. 2d, dashed black circles). This diffuse density was separated from the rigid dimer structure in the model by a distance consistent with flexible HAT-like domains. We interpret the lack of a single stable density associated with the C-terminal HAT domain in these 2D classes to reflect the extreme conformational flexibility of the segment joining the domains. This work provided a framework to build a model of the full length OGA-L from a combination of our high-resolution cryo-EM model of the catalytic-stalk dimer and an Alphafold2 prediction of the HAT-like domains docked into the low-resolution density (green) (Fig. 1e). In this model, the HAT-like domains are defined, and the densities correspond to the size and fold predicted from Alphafold2 and homologous crystal structures ^21^. However, the resolution was insufficient to identify sidechains or properly orient the Alphafold2 predicted HAT-like domains into the density of the map. Overall, the model provides strong evidence for highly flexible linkage between the HAT-like domains and the rigid arm-in-arm dimer comprising the catalytic and stalk domains.

Alphafold2 predictions and our cryo-EM map show a highly conformationally flexible structure. To further evaluate the flexibility of the OGA-L C-terminal domain, the program FastFloppyTail^54^ was used to produce an unbiased ensemble of models of OGA-L (Fig. 2b, green). The dimensions of the distribution of the HAT-like domains (Fig. 2b) and the low resolution OGA-L model (Fig. 2a) reveal the highly flexible nature of the molecule and correlates well the diffuse cloud of densities that span a cone shaped area of approximately 70 Å by 160 Å (Fig. 2b,c).

It is well established the enzymatic activity of OGA-S is significantly attenuated compared to OGA-L (Supplementary Fig. 1a, right) ^55,56^. To explore possible structural basis for this difference we compared the Alphafold2 predicted structure of OGA-S with our combined model of OGA-L (Supplementary Fig. 3). Overall, the catalytic and stalk regions of OGA-L and OGA-S are very similar, however OGA-S lacks a helix present in the stalk region of OGA-L that interacts in trans between the two monomers (Supplementary Fig. 4, purple). In addition, OGA-S terminates in a short alpha helical segment that could alter stalk movement (Supplementary Fig. 3e,f, orange). Thus, the HAT-like domain may contribute to the observed elevation in enzymatic activity of OGA-L by acting through this helical segment in the stalk domain.

### Histone binding Features of *O-*GlcNAcase and purified HAT domains

**Fig. 3:**
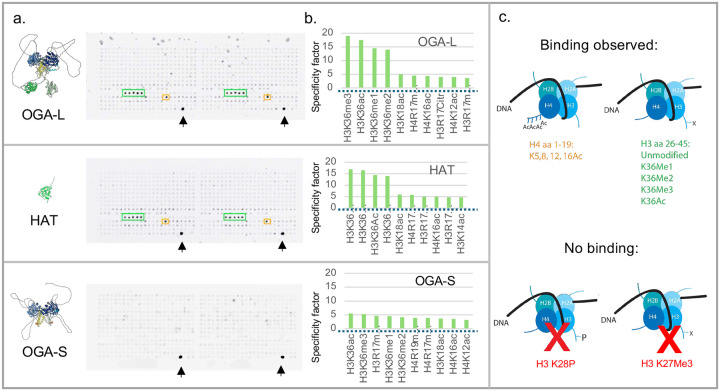
OGA-L and HAT domains bind to a shared subset of modified histones on a Modified Histone Array. **a**, Left, Alphafold2 models of OGA-S, HAT-like domain, and OGA-L, colored by domain: catalytic domain, dark blue; stalk, yellow; linker and HAT-like domain: green and corresponding assayed Histone Arrays probed with those domains (**a**, Right). The arrays are analyzed in duplicate and representative quantification of these arrays is shown in **b**. The arrays were probed and processed as described in materials and methods and the peptides that bound most selectively are highlighted in green and orange boxes. The arrays were probed with each protein and with two independent antibodies to identify peptides binding with highest selectivity. Those peptides showing highest selectivity indices are shown in **c** top. Peptides with lowest selective binding are shown in **c** bottom. Arrows indicate the positive controls for the histone array showing the maximum signal possible by this detection method.

The long isoform of mammalian *O-*GlcNAcase (OGA-L) was previously suggested to exhibit histone acetyl transferase when purified from Hela extracts and was termed NCOAT for Nuclear Cytoplasmic *O-*GlcNAcase and Acetyltransferase ^57,58^. These authors argued for an interaction with Histone H4 and acetylation through a zinc-finger motif unique to MYST domain HATs. However, other studies have demonstrated that the “Pseudo-HAT” domain of *O-*GlcNAcase lacks important residues for binding to Acetyl-CoA and exhibits no HAT activity *in vitro* ^21,55^. The overall structure of the HAT domain is like that of other histone binding domains. The similarities in structure of the *O-*GlcNAcase pseudo-HAT domain to other protein histone acetyltransferases prompted us to examine the ability of *O-*GlcNAcase and HAT domains to bind to an unbiased collection of modified histone tails. The short variant of OGA provided an excellent biologically relevant control lacking the HAT domain. We chose the MODified^™^ histone peptide array which interrogates 384 unique histone modifications in duplicate. The high density of modified histone tails present on the array allow detection of a wide range of binding affinities. We expressed and purified the long and short isoforms of the human *O-*GlcNAcase and the isolated HAT domain with several epitope tags. These isoforms were then used to probe multiple arrays. Both the full-length OGA long form and the isolated HAT domain reproducibly bound to a small subset of histone tails present in duplicate on the arrays (Fig. 3a and Supplementary Fig. 5). The short *O-*GlcNAcase isoform lacking the HAT domain showed no obvious selective binding (Fig. 3a). In contrast, the OGA-L variant and the HAT domain bound with highest selectivity to acetylated H4 aa 1-19 and H3K36 26-45 peptides. The selectivity of binding was highest for singly modified H3K36 variants: K36^Me1^, K36^Me2^, K36^Me3^ and K36^Ac^ (Fig. 3a, green box). Multiply acetylated (H4K^5,8,12,16Ac^) also bound to both OGA-L and the HAT domain (Fig. 3a, orange box). Figure 3b shows the binding “selectivity factor” for binding to each array as defined by the Array Analyze Software^™^ used to quantify the commercial arrays. Only histones peptides with single modifications were included in the “selectivity factor” analysis. The modified H3K36 variants were the only singly modified peptides to reproducibly show a selectivity factor above a threshold of 5. The detection of the binding was independent of the antibody used to detect the binding on multiple arrays (His6 tag or C-terminal tag). The overlap in observed binding interactions between the full-length OGA molecule and the isolated HAT domain was also striking with the highest selective binding interactions detected being identical. These modifications on H4 and H3 are all associated with transcriptional activation and open chromatin domains. Those interactions shared by the HAT and OGA-L domains were (in order of specificity factor): H3K36Me3 > H3K36Me2 > H3K36Ac > H3K36Me1 > H3K36unmodifed> H4 1-19 K5,8,12,16Ac > H4K16Ac > H3R17Me2 > H3R17Citr > H3K18A > H4R17Me2. Intriguingly, negligible binding was observed with H3K16–35 S28^P^ associated with histone stability or H3K27Me^3^ modified peptides associated with transcriptional repression. These histone modifications are generally mutually exclusive in mammalian genomes ^59^.

### The Structural Features of OGA-L suggest a mode of interaction with modified histone tails in Nucleosomes

**Fig. 4:**
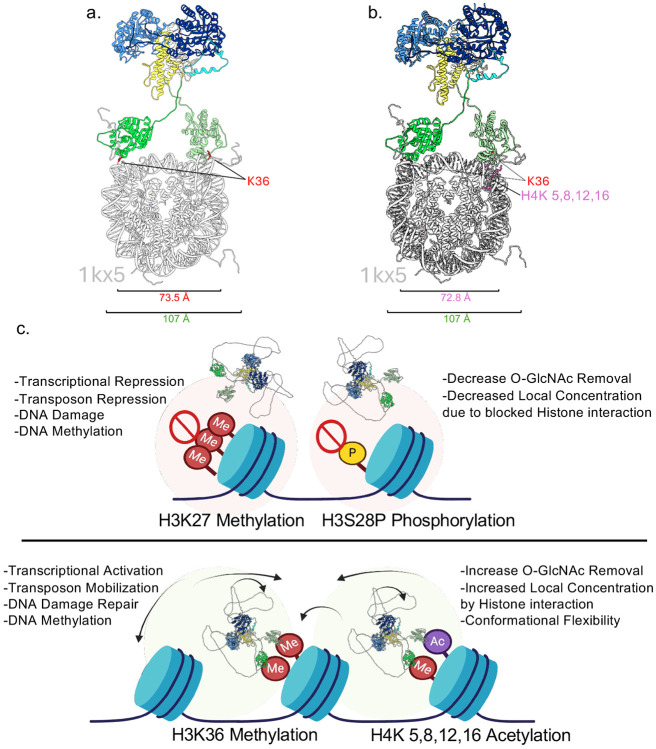
Model of OGA-L bound to histone tails of the Nucleosome. **a**,**b,** Potential binding sites of the HAT-like domains to nucleosomes highlighting proximity and spacing of the H3K36 residues (**a**) as well as the H3K36 and H4K 5,8,12, and 16 residues (**b**) in the nucleosome (PDBID:1kx5: grey). OGA is colored by domain: catalytic domain, dark blue; stalk, yellow; linker and HAT-like domain: green. The distance between K36 residues (red) is red 73.5 Å, and the total distance between HAT densities is 107 Å (green). The distance between K36 residue and the H4K 5,8,12,16 is 72.8 Å (pink). **c,** The binding characteristics and structural features of OGA-L have important biological implications. The OGA-L HAT domain shows no binding to modifications associated with transcriptional silencing such as H3K27^Me3^ linked to polycomb suppression. OGA-L HAT binding to histone modifications such as H3K36^Me^ and acetylated H4 tails would facilitate recruitment to sites of active transcription and DNA repair. The structural features identified for OGA-L are likely to increase the local concentration of the OGA-L and allow flexible movement of the catalytic domain to facilitate *O-*GlcNAc removal from proteins in proximity. The OGA model is shown with unstructured linkers added from the Alphafold2 colored by domain: catalytic domain, dark blue; stalk, yellow; linker and hat-like domain green.

Taken together our findings suggest that the HAT-like domain of OGA-L is tethered to the catalytic-stalk dimer by a flexible linkage. The identification of the modified histones which bind to the HAT-like domain and our cryo-EM structure allow us to model how this interaction might occur physiologically (Fig. 4). The histone tails of the nucleosome are known to be highly flexible appendages to the core histone octamer ^60^. The OGA-L cryo-EM structure is shown in juxtaposition with the position of the two H3K36 residues that are methylated or acetylated on the X-Ray structure of the nucleosome (1kx5) (Fig. 4a). Remarkably, the distance between the two modified H3K36 histone tails (~75 Å) could easily be bridged by the flexible HAT-like domains in our cryo-EM structure. Similarly, the distance between the two H4K 5,8,12,16Ac sites on nucleosomes (~73 Å) could each be recognized by the HAT-like domains in our cryoEM model (Fig. 4b). The intrinsic flexibility of the OGA-L molecule would also allow substantial conformational flexibility of the tethered catalytic-stalk dimer upon binding to the modified nucleosome. The potential biological implications of these findings are summarized in a model in Fig. 4c. OGA-L binding was not observed with either H3K27 methylation or H3S28P histone modifications. These modifications correlate with closed chromatin states associated with transcriptional silencing, transposon repression, and DNA damage and methylation. In contrast, the histone modifications to which OGA-L binds are linked to open chromatin conformations. These modifications are associated with transcriptional activation, transposon mobilization, DNA damage repair and DNA methylation/demethylation. Thus, the histone interactions observed would promote increased *O-*GlcNAc removal by increasing the local concentration of the OGA-L enzyme in open chromatin and the conformational flexibility of the enzyme would allow *O-*GlcNAc removal from targets in proximity such as the CTD domain of RNA Polymerase II or components of the DNA damage response. Like the 55 heptad repeats comprising the CTD domain of RNA Polymerase II, most known OGT targets are *O-*GlcNAc-modified in intrinsically disordered regions ^9^.

## Discussion

### The enzymes of *O-*GlcNAc cycling are conformationally flexible multi-domain proteins

Like the TPR domain of OGT, OGA-L exhibits substantial conformational flexibility that may relate to its enzymatic function and cellular function. Although considerable progress has been made in defining the structure of these enzymes, the details of the molecular recognition of protein targets remain poorly understood. These two enzymes act antagonistically by adding and removing *O-*GlcNAc from thousands of cellular proteins and therefore are likely to have both diverse and versatile modes of target recognition ^9^. The spring-like flexibility of the OGT TPR domain may provide a scaffold for binding of disordered peptides as was observed with a recent structure of an OGT-OGA complex ^45^. This structure of the dimeric OGT associated with *O-*GlcNAcase (a substrate for OGT) demonstrates that the inner portion of the TPR domain forms extensive contacts with the intrinsically disordered regions of the *O-*GlcNAcase monomer. These authors have proposed that this complex is autoinhibitory; neither enzyme is enzymatically active when associated in this fashion. It is of considerable interest to determine whether interaction between the two enzymes of *O-*GlcNAc may be a part of their intracellular regulation. Here we solved the structure of full-length *O-*GlcNAcase, OGA-L, by cryo-EM methods. The observed structural features of this molecule provide important new insights into how it may function.

### Cryo-EM of Full-length Human *O-*GlcNAcase

The cryo-EM structures of full-length *O-*GlcNAcase reported here significantly adds to existing knowledge of the organization of the enzyme (Fig. 1). Previous crystal structures were obtained by truncation of the HAT-like domain and disordered regions to obtain crystal structures of the catalytic-stalk dimer. Thus, substantial segments including the entire unstructured C-terminal domains were excluded. Here we used full-length *O-*GlcNAcase molecule to generate a physiologically relevant cryo-EM map. Intriguingly, the overall cryo-EM structure of the catalytic-stalk dimer differs notably with that of the crystal structures and intrastrand helices holding the catalytic-stalk dimer together can be visualized ^18-20,61^. The cryo-EM structure also allows resolution of the TIM-barrel structure organizing the catalytic center of the molecule and individual side chains can be detected. The position of active site residues is not dramatically different from those observed in the crystal structures. As the crystal structures demonstrated, the catalytic domain of OGA is formed as an “arm in arm” dimer in which the catalytic domain of one monomer associates with the stalk domain of the other monomer ^18-20,61^. This organization creates a substrate-binding cleft and residues on the cleft surface could potentially afford extensive interactions with peptide substrates ^18-20,61^. Our cryo-EM map of full-length OGA-L approximates the existing crystal structures confirming this dimeric organization and the overall conformation of the molecule. However, the cryo-EM map deviates from crystal structures in several important ways. First, the presence of the additional flexible regions and HAT-like domain in the OGA-L construct likely resulted in the differences observed between our cryo-EM map and crystal structures, particularly the decreased packing within the catalytic-stalk dimer interface. This may explain why Alphafold2 predictions better match the cryo-EM structure than existing crystal structures. Second, we observe an asymmetry between the two chains of the OGA-L molecule with a displaced helix visualized on one of the chains. This helical segment is positioned so that it could alter either the interaction with substrates or catalytic efficiency.

### OGA-L has an intrinsically disordered flexible linkage between catalytic and Histone Binding Domains

The cryo-EM structure of the human *O-*GlcNAcase also provides evidence for substantial flexibility between the intrastrand dimer of the catalytic and stalk domains and the pseudo-HAT domains. The 2D classes show evidence of diffuse electron density corresponding to various positions of the Histone-interacting domains. These segments are likely to exhibit substantial flexion with respect to stable dimer of the catalytic and stalk domains as predicted by Alphafold2. A likely explanation for this diffuse density is flexion of the HAT domains relative to the highly stabilized catalytic-stalk dimer. The overall dimensions suggest that the intact molecule may span roughly 70 Å from the Histone interacting domain to each catalytic domain. The spacing of the ordered catalytic-stalk dimer and the diffuse density corresponding to the HAT-like domain is consistent with the predicted length of the intrinsically disordered linker region. Thus, full length *O-*GlcNAcase is not a rigid dimeric molecule but consists of a highly stable catalytic-stalk dimer linked to mobile histone interacting domains by a flexible linker. This structural flexibility may have a significant effect on functioning of the enzyme as described below.

### HAT domain binding to H4 and H3 Histone tail modifications

Both the full-length *O-*GlcNAcase and the purified HAT domain were used to probe modified histone arrays allowing a reproducible and unbiased assessment of histone tail interactions. This analysis demonstrated that both the HAT domain and the full-length *O-*GlcNAcase bound selectively to only a small overlapping subset of histone tail modifications. The overlap between the observed binding with the isolated HAT domain and full-length *O-*GlcNAcase was striking. In each case only variants of histone H3 and H4 bound with significant specificity. No selective binding was seen with the short *O-*GlcNAcase isoform lacking the HAT domain. Little if any selective binding was observed with the vast majority of the 384 peptides on the arrays including H3K27 and H3K28 variants. The most selective binding was observed with di-and tri-methylated H3K36 containing peptides. Significant binding was also observed with H4K5,8,12,16Ac. Intriguingly, variants of this H4 peptide were previously shown to bind to *O-*GlcNAcase in a previous analysis although this was attributed to substrate recognition by a “HAT” activity ^57^. Both H3K36 and H4 histone modifications which bind to the HAT domain of *O-*GlcNAcase selectively are associated with transcriptional activation and open polynucleosome conformations ^59,62^. The histones identified also play roles in other key nuclear events such as DNA repair, transposon repression/mobilization and DNA methylation as detailed below.

### *O-*GlcNAcase-binding Histone modifications have Important Roles in Transcriptional Activation and Transposon silencing

The long isoform of *O-*GlcNAcase is localized in both nucleus and cytoplasm ^17^. Here, we have shown that OGA HAT domain also binds selectively to methylated and acetylated forms of H3K36Me^1-3^ and H4K^5,8,12,16ac^. The small number of modified histones that selectively bind to OGA via the HAT domain provides an important clue as to how *O-*GlcNAcase functions at the level of chromatin structure.

The H3K36Me^1-3^ and H4K^5,8,12,16ac^ histone tail modifications are both thought to induce open nucleosome conformations required for active transcription ^35,59^. However, they may induce these open nucleosome confirmations by different mechanisms ^62^. This open conformation of chromatin is associated with several important biological processes. It is also intriguing that no specific *O-*GlcNAcase binding was observed with H3K27Me^3^ or H3K28^P^ containing peptides. These repressive modifications are associated with transcriptional silencing during the cell cycle, X-chromosome inactivation, and the silencing of retrotransposons ^23,59,63^. The H3K27Me^3^ mark is a well-established mode of transcriptional repression termed polycomb repression. OGT is associated with the polycomb repressive complex (PRC2) in *Drosophila* and in mammals ^24,30-33,38^. We, and others, have shown that *O-*GlcNAc levels are very high at H3K27Me^3^ regions of the genome associated with polycomb repression ^30-33,38^. In general, the H3K36 Me^1-3^ modifications and H3K27Me^3^ modifications are mutually exclusive across the genome. However, in some cases, these histone marks are both present at the promoters of developmentally regulated genes termed “bivalent” promoters ^63^. The antagonism between the H3K36 Me^1-3^ modifications and H3K27Me^3^ modifications is a well-documented aspect of transcriptional regulation. Taken together, our finding suggests that *O-*GlcNAcase becomes concentrated in regions of open chromatin enriched in H3K36 Me^1-3^ and H4K^5,8,12,16ac^. The H3K36 modification is associated with actively transcribing regions of the genome where PolII is fully engaged. Many transcription factors including the carboxy-terminal domain (CTD) of RNA Pol II are *O-*GlcNAc-modified and may be subject to *O-*GlcNAc removal at these actively transcribing regions. In particular, the ‘CTD-code’ of sequential phosphorylation events leading to transcription requires *O-*GlcNAc removal from the CTD-domain of Pol II ^28,39,40,64,65^. Once *O-*

GlcNAc is removed, phosphorylation of residues within the heptad repeats of the Pol II CTD is associated with transcriptional initiation, elongation and termination ^26,28,39,40^. We have previously suggested that this series of posttranslational events may be required for proper transcriptional regulation^28,39,40,64,65^.

The transcriptional repression of retrotransposons in mammals is a complex and redundant means of maintaining genome integrity ^66^. Recent evidence suggests that *O-*GlcNAc cycling is an important contributor to repression of transposable elements such as LINE-1 ^23^. OGT was shown to be uniquely recruited to sites of LINE-1 insertion by interacting with the Zn-finger protein KAP1 at genomic regions subject to DNA methylation. When *O-*GlcNAcase was artificially recruited to these elements using Cas-9 fusion, LINE-1 was actively expressed. These findings suggest that *O-*GlcNAcase may normally be restricted from chromatin domains established during transposon silencing which include the H3K27Me^1-3^ modification.

### *O-*GlcNAcase and DNA Damage repair

The H3K36Me^1-3^ and H4K^5,8,12,16ac^ histone tail modifications which selectively bind to OGA are each associated with the process of DNA damage repair ^35,59,67,68^. Intriguingly, *O-*GlcNAcase is also required for DNA repair ^25,34,69^. We and others have shown that loss of *O-*GlcNAcase in actively growing cells triggers an increase in DNA double strand breaks ^34,69^. In addition, loss of OGA induces DNA damage signaling through ATM/ATR signaling and CHK1/2 stabilizes OGT creating an autoregulatory feedback loop ^69^. Both OGT and OGA are normally recruited to sites of DNA damage and many DNA repair factors are *O-*GlcNAc modified ^34,69,70^. A recent paper showed that the HAT-domain of *O-*GlcNAcase was required for recruitment to sites of DNA damage ^34^. These authors proposed a model by which *O-*GlcNAcase is recruited to sites of DNA damage by its HAT domain to remove *O-*GlcNAc from molecules such as Ku70/80 mediating the process of non-homologous end joining (NHEJ). Thus, our findings regarding the HAT domain histone-binding characteristics are consistent with this model of OGA recruitment to sites of double strand DNA breaks.

### A Model of *O-*GlcNAcase and Structure-function Relationship of the HAT domain

The conformational flexibility between the two more ordered domains of *O-*GlcNAcase highlighted by the cryo-EM structure is likely to be an important contributor to the functioning of the enzyme. The 2D classes clearly show a distribution of the flexible pseudo-HAT domain relative to the stabilized cross chain dimer of the catalytic domain (Fig. 2c). The intrinsic flexibility of the *O-*GlcNAcase may be important for the functioning of the enzyme in mediating events associated with removal of *O-*GlcNAc in proximity to modified H3K36 histone domains. Histone octamers contain two copies of the H3K36 site which can be modified. The crystal structure of the histone octamer (pdb:1KX5) shows that these two H3K36 sites are separated by approximately 70 Å which suggests that the highly flexible *O-*GlcNAcase HAT domains could potentially bind simultaneously to the two modified H3K36 sites on the nucleosome (Fig. 4). It is also possible that other combinations of histone tail modifications could be recognized by OGA-L that did not emerge from our histone array analysis.

In summary, our findings suggests that *O-*GlcNAcase binding to modified H3K36 and H4 histone tails would allow a flexible tethering and increased local concentration of the catalytically active enzymes in chromatin regions enriched in these histone modifications (Fig 4). These modifications are both associated with open chromatin. In contrast, *O-*GlcNAcase shows no selective binding to H3K27 methylation or H3K28 phosphorylation sites associated with transcriptional repression and closed chromatin. Thus, histone domains containing these modifications would have a reduced local concentration of *O-*GlcNAcase relative to H3K36-containing domains. In general, domains rich in methylated H3K36 and methylated H3K27 are mutually exclusive in eukaryotic genomes suggesting that the *O-*GlcNAcase concentrations in those regions may vary dramatically. The highly selective binding of *O-*GlcNAcase to H3K36Me^1-3^ is consistent with the known roles for *O-*GlcNAcase in H3K36 Me^1-3^ -mediated processes such as transcriptional activation, transposon regulation and DNA damage repair. These evolutionarily conserved histone modifications play a key role in development, stem cell biology and differentiation. Our structural work has identified OGA-L as a potential ‘reader’ of this aspect of the histone code.

## Material and Methods

### Expression and Purification of Epitope tagged *O-*GlcNAcase and subdomains

#### Protein expression and purification

Each of the OGA fragments and subdomains were cloned into the pBAD/HisA plasmid in frame with the epitope tags after codon optimization by GeneART Gene synthesis services (Invitrogen). Each of the resulting plasmids were sequenced to verify the coding sequence expected. These fragments and their corresponding sizes are shown in (Supplementary Fig. 1). The protein and DNA sequences for each of the fragments is also shown in (Supplementary Fig. 1). The pBAD/HisA OGA plasmids were transformed in the TOP-10 bacterial strain, which contain the *araBAD* promoter. For each protein preparation, the following steps were carried out. On day 1, a 5 mL LB-Carb culture was grown at 37°C overnight shaking at 200 rpm. On day 2 the cultures were pelleted at 4700 rpm for 5 minutes and the supernatant removed. The pellet was resuspended in 500 mL or 1 liter of Terrific Broth (Thermofisher) with Carbenicillin added (final carbenicillin concentration is 50 ug/mL). Cultures were grown at 37°C shaking at 200 rpm until the OD_600_ reached 0.8 (usually about 3.5 to 4 hours for 1 L culture) and cooled to room temperature for 30 minutes. Arabinose (final concentration of 0.02%) was added to the culture to induce protein expression and cultures were incubated at 20°C overnight with shaking. On day 3, the culture was centrifuged at 4700 rpm at 4°C for 10 minutes and the supernatant was removed. The pellet was flash frozen in liquid nitrogen or on dry ice. The pellets then subjected to the lysate preparation step immediately or stored at −80°C.

#### Lysate preparation

The *E. coli* cell pellets were thawed on ice, then suspended in 25 mL of Lysis buffer. The lysis buffer consisted of 50 mM NaPhos pH 7.8, 500 mM NaCl, 10 mM imidazole, and 0.1% Tween-20 to which 0.2 mM PMSF has been added fresh. Lysate was transferred to a 50 ml conical tube. The mixture was sonicated using a flat tip 3 X 30-45 seconds on ice. β-mercaptoethanol (BME) was added to a final concentration of 5 mM and the mixture was inverted several times to mix. The resulting mixture was centrifuged at 35K rpm for 30 min- 1 hours at 4°C. The supernatant was the cellular lysate containing the expressed protein. After ultracentrifugation at 100,000 X g, the supernatant was transferred to a 50 mL conical tube. Talon resin (Takara Bio) was added directly to the supernatant. OGA proteins were allowed to bind for at least 1 hour at 4°C with spinning or rocking. For 1-liter cultures, 2 mL of the Talon resin mixture was added. The beads were pelleted by spinning very gently at 500 rpm for 2 min at 4°C. The unbound fraction was removed and full length OGA or subdomains were present on the beads. Wash buffer (25 microliters) was added to the beads (50 mM NaPhos pH 7.8,500 mM NaCl, 30 mM imidazole, 5 mM BME and shaken to resuspend the beads. The beads were pelleted by spinning very gently at 500 rpm for 2 min at 4°C. This was repeated 3 times. The bead and liquid slurry were applied to a 5 mL Pierce Centrifuge Column (Product #89897) and spun gently at 500 rpm for 2 min. at 4°C to remove the remaining Wash Buffer. The pellet was treated with 1 mL of Elution buffer (50 mM NaPhos pH 7.8, 500 mM NaCl, 300 mM imidazole) and rocked at 4°C for 5 minutes. To collect purified OGA, remove bottom cap, place in new 15 mL conical and spin gently at 500 rpm for 2 min. at 4°C. Elution was repeated 3 times to maximize protein recovery. Eluted proteins were stored at 4°C or directly used for histone array experiments. Protein staining and Western blotting of the eluted proteins suggested that the epitope tagged proteins corresponded to the appropriate molecular mass for each of the variants or full length OGA-L molecules.

#### Protocol for *O-*GlcNAcase binding to Modified histones using Active Motif Modified Array Labeling Kit

Histone arrays were probed with full length OGA, the HAT domain, or the short isoform lacking the HAT domain using a minor modification of the procedure described in the manufacturers protocol (Active Motif MODified^™^ Histone Peptide Array). All values presented are based on probing 3 or more histone arrays using the His6 epitope tag antibody. To verify specificity, arrays were also probed with the C-term and Xpress epitope tag antibodies (Supplementary Table 2). The C-term antibody gave results very similar to the His6 tag, but the Xpress tag antibodies bound to the histone array with less specificity and was therefore excluded from the analysis. Briefly, arrays were blocked with 3 mL AM2 Blocking Buffer provided in the Active Motif Kit at RT for 1 hour with shaking. Arrays were washed three times with supplied wash buffer. For each protein probed. 100 nM of protein was added in 3 mL of 20 mM Tris pH 7.5, 1 mM DTT, 0.5 mg/mL BSA and incubated at RT for 1 hour with shaking. Blots were washed 3 X prior to antibody addition. Both Anti-His6 antibody and the Anti-C-myc antibody were added at 1:2000 in 3 mL total volume of the AM2 buffer and incubated at 4°C overnight. After 3X washing, anti-mouse-IR-800 antibody at 1:2000 in 3 mL AM2 was added and incubated at RT for 1 hours with shaking. After washing two additional times, imaging was performed using the LI-COR Odyssey DLx I LICORbio imaging system. Arrays were analyzed using the Active Motif Array Analyze Software as described by the manufacturer (Active Motif). The software program analyzes the intensity of spot interactions from the MODified Histone peptide array and generate a graphical analysis of the histone peptide modification interactions. With the Array Analyze Software, information about spot intensity, averages and errors were saved in Micosoft Excel-compatible files allowing for our individual analysis. Output from the Active Motif Array Software is presented as “Specificity Factors”. This is a background subtracted graph of the 10 modifications with the greatest specificity factors. While all modifications have been accounted for in determining the specificity factor, only singly modified peptides representing the 10 modifications with the highest specificity factor values are graphed.

#### Expression and Purification of Full-Length Human OGA Protein for cryo-EM

All constructs were codon optimized for expression in *E. coli.* The full-length of human OGA protein was subcloned into a pBAD vector with an N-terminal His6-tag as described above. The plasmids were transformed into Escherichia coli TOP10 cells. Cultures were grown at 37 °C in Luria–Bertani (LB) medium until the optical density at 600 nm (OD600) reached 0.8. At this point, the culture was cooled to 16 °C and protein expression was induced with 0.4 mM arabinose. After 16 hours of induction, cells were harvested and lysed at 4 °C using an ultra-high-pressure cell disruptor. The lysate was subjected to affinity purification on Cytiva HisTrap columns, with the target protein eluted in a single step using a buffer containing 250 mM imidazole. The protein was further purified by size-exclusion chromatography (Enrich SEC 650 10/300 MM, Bio-Rad) in a buffer containing 20 mM Tris, 150 mM NaCl, and 0.5 mM Tris(hydroxymethyl)phosphine (THP). The purified protein samples were flash-frozen in liquid nitrogen and stored at −80 °C. The resulting protein was judged to be > 95 pure by Coumassie brilliant blue staining.

#### *O-*GlcNAcase Activity Assays

*O-*GlcNAcase assays on bacterially produced recombinant OGA were performed essentially as described previously ^55,71^. For assays in OGA knockout (KO) MEFs (Keembiyehetty et al., 2015), cells were lysed in RIPA buffer (10 mM Tris-HCl, 150 mM NaCl, 1% Triton X-100 [v/v], 0.5% sodium deoxycholate [w/v], 0.1% sodium dodecyl sulfate [w/v], and protease inhibitors; pH 7.5). The lysates were vortexed and centrifuged at 14,000 rpm for 20 minutes at 4 °C.mFor the enzymatic activity assay, 30 μg of clarified lysate, with or without purified OGA, was added to a reaction mixture containing 200 μM fluorescein di(N-acetyl-β-D-glucosaminide) (FDGlcNAc) and 50 mM N-acetylgalactosamine (GalNAc) in 50 mM citrate/phosphate buffer (pH 6.5). Reactions were incubated in the dark at 37 °C with shaking at 100 rpm for 30 minutes. The reactions were quenched by the addition of sodium carbonate (Na_2_CO_3_) to a final concentration of 400 mM. Fluorescence was measured in 1-s intervals at the excitation wavelength of 485 nm and at the emission wavelength of 535 nm on a Wallac 1420 fluorometer (PerkinElmer Life Sciences).

#### SDS-PAGE and Western Blot analysis

Protein lysates were mixed with Laemmli buffer and boiled at 95 °C for 5 minutes prior to separation by SDS-PAGE. Samples were resolved on NuPAGE 4–20% Bis-Tris gels (Invitrogen) and visualized using Coomassie blue staining or Western blotting. For Coomassie staining, the gels were incubated in PageBlue Protein Staining Solution (Invitrogen) on a rocking shaker for 1 hour, followed by destaining with water for an additional hour. For Western blot analysis, proteins were transferred onto a nitrocellulose membrane and blocked for 45 minutes with 5% [w/v] nonfat milk in Tris-buffered saline containing 0.1% [v/v] Tween 20 (TBS-T). Membranes were incubated overnight at 4 °C with primary antibodies diluted in milk/TBS-T with gentle agitation. After primary antibody incubation, the membranes were washed three times with 10 mL of TBS-T for 10 minutes per wash and subsequently incubated with fluorescent-conjugated anti-mouse secondary antibodies (LI-COR) at a 1:10,000 dilution for 1 hour at room temperature. Three additional washes with 10 mL of TBS-T for 10 minutes each were performed before imaging the blot on an Odyssey Fc imager (LI-COR).

#### Cryo-Electron Microscopy Sample Preparation, Data Collection, Processing and Refinement

Purified OGA protein was diluted to a concentration of approximately 0.5 μg/μl in 20 mM Tris and 150 mM NaCl. A 3μl aliquot of the protein solution was applied to a glow-discharged grid (30 s glow discharge in a glow discharge cleaning system, Pelco easiGlow (Ted Pella, inc.)). Following a 15-s incubation at >95% relative humidity, excess protein was removed by blotting for 5 s. The grids were then plunge-frozen into liquid ethane using a Leica EM GP2 plunge freezer (Leica Microsystems). Vitrified grids were stored in liquid nitrogen before examination using cryo-electron microscopy (cryo-EM).

The cryo-EM data were collected using a Titan Krios G3 microscope (Thermo Fisher), operating at 300 kV, equipped with a K3 detector (Gatan). 1,737 images were collected at a magnification of 105,000x with calibrated pixel size of 0.53 Å, nominal defocus range of 0.3 to 2.4 μm, 50 frames, and 67 e-/Å2 electron exposure per movie.

Images were processed using cryoSPARC v4.6.0 (Supplementary Fig. 1b) ^51,52^. Images were corrected using Patch Motion correction and Patch CTF estimation in cryoSPARC ^51,52^. Particles were selected using the blob picker, extracted with a 280-pixel (1.2114 .2518 Å/pixel) or 440 pixel (1.2045 Å/pixel) box size, and subsequently pruned using iterative 2D classification. Initial alignment was performed using the *ab initio* reconstruction job. Refinement was performed using iterative heterogeneous refinements, non-uniform refinements, 3D classifications, and local refinements using C2 or C1 symmetry. Masks for local Refinements were generated in Chimera ^72^.

Maps generated in cryoSPARC were post-processed using deepEMhancer to sharpen protein densities and sharpened in cryoSPARC as a cross-comparison for model building ^73^.

The initial model of the OGA dimer was produced using Alphafold2 multimer, which was fit into generated maps in UCSF Chimera ^72,74^. Iterative model refinement was performed using Rosetta v2021.16, Phenix v1.29.1-4487, Gaussian mixture model based atomic model refinement in EMAN2 v2.99.66, and Coot v0.9.8.92 then assessed using MolProbity and Phenix ^74-79^. The cryo-EM data collection, final refinement, and validation statistics for the model are presented in Supplementary Table 3. Structural analysis, measurements and figures were prepared in Chimera 1.15 and ChimeraX 1.2.5 ^72^. Interfacial area and hydrogen bonding was calculated using the PDBePISA server ^53^.

Motion of the disordered region, residues 694-712 (PIDGANDLFFQPPPLTPTS), between the stalk and HAT-like domain was modelled using the PyRosetta based algorithm, Fast^54^. 600 structures were generated, and 75 structures were used to display the ensemble in Figure 2B. From this unbiased distribution, subsets of this ensemble were selected which best fit into the 2D class densities in Figure 2C.

## Supplementary Material

Supplement 1

## Data Availability

Structural data supporting findings in this study have been deposited in the Protein Data Bank (PDB) and the Electron Microscopy Data Bank (EMDB). The accession codes of the cryo-EM maps and accompanying atomic models have been provided for: (1) OGA-L Catalytic Dimer (PDB ID: 9EN2, EMDB-49293), 2) A-chain of the OGA-L Catalytic Dimer (PDB ID: 9EN4, EMDB-49294), 3) B-chain of the OGA-L Catalytic Dimer (PDB ID: 9EN5, EMDB-49295), 4) OGA-L Dimer (EMDB-49296), 5) OGA-L Catalytic Dimer, A-chain extra density (EMDB-49297). Antibodies employed in this study can be found in Supplementary Table 2. *Additional data compared in this study from the protein databank: Alphafold2 Multimer, PDB ID: 5M7R, PDB ID: 5VVO, PDB ID:5UHK, PDB ID: 5M7S, PDBID:5UN9, PDB ID:5UHL, PDB ID: 5M7T, PDB ID: 5UHO, PDB ID: 8P0L, PDB ID: 9BA8, 9BA9, PDB ID: 1KX5.*

## Code availability

Custom codes were not central to the conclusions of the paper.
