# Supplementary material for: Human O-GlcNAcase catalytic-stalk dimer anchors flexible histone binding domains
Source: Res Sq. 2025 Apr 1:rs.3.rs-6197257. Preprint. [Version 1] doi: 10.21203/rs.3.rs-6197257/v1 (PMC12747287; doi:10.21203/rs.3.rs-6197257/v1)
Supplement: Supplement 1 [file NIHPPrs6197257v1-supplement-1.pdf]

Supplementary Figures and Legends:

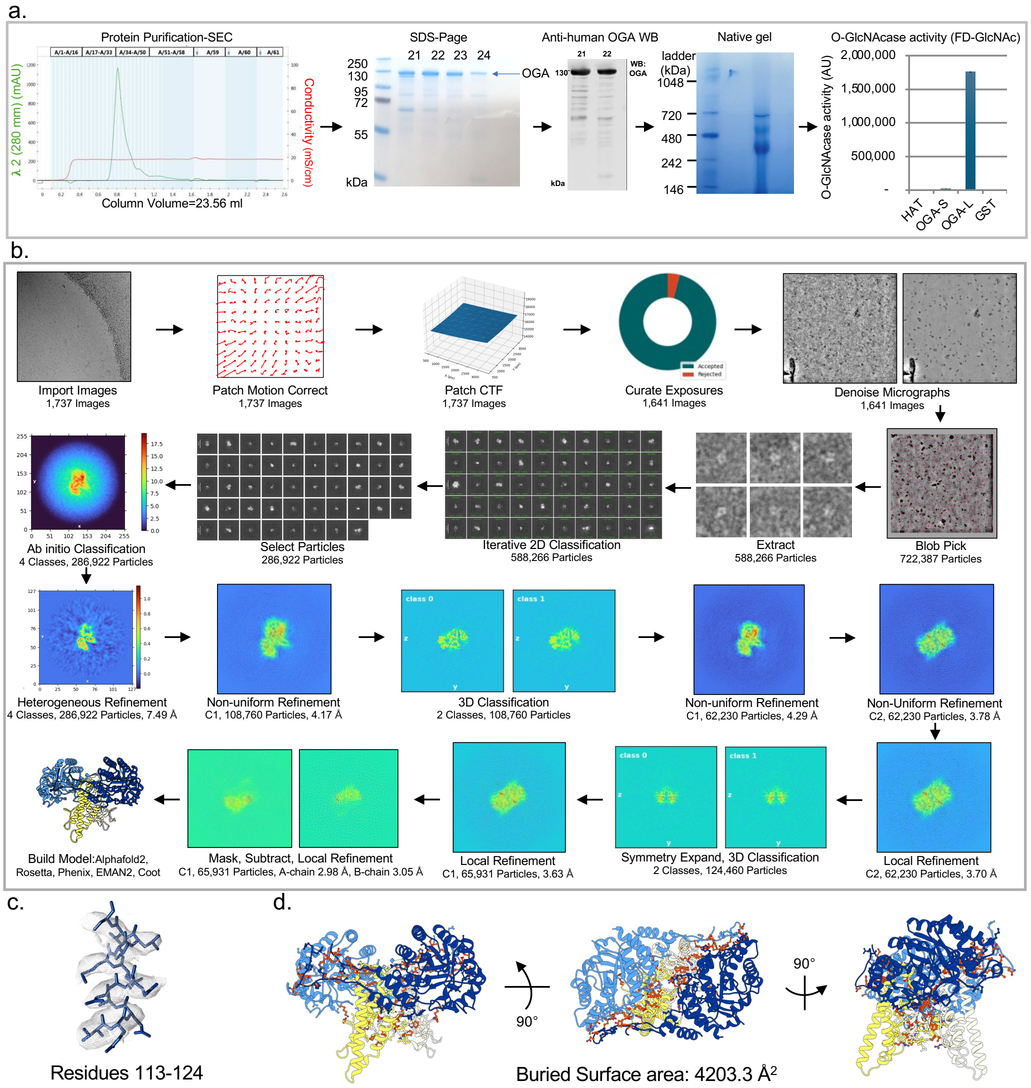

Supplementary Fig. 1: Purification, characterization, and workflow for determining the cryo-EM structure of the OGA-L dimer. a, Extracted protein was affinity purified using a His-column followed by size exclusion chromatography (left). Pooled fractions were analyzed by SDS-PAGE and western blotting using anti-human OGA. Migration of the purified protein on a native gel was consistent with dimerization of the protein with smaller amounts of higher oligomers. The activity of the purified OGA-L was determined using a fluorogenic substrate and compared to the expressed HAT domain, the OGA-S short *O*-GlcNAcase isoform, and a His-tagged GST control (right). **b,** An outline of cryo-EM data processing. Each dataset followed a standard workflow including patch motion correction, patch CTF correction, and exposure curation. Micrographs were denoised, then blob picker was used to select particles and subsequently extracted. Particles were iteratively 2D classified to remove junk and then aligned through *ab initio* reconstruction. The selected *ab initio* model was used for heterogeneous

refinement, subsequent non-uniform refinement, and 3D classification using C1 symmetry. A single class was then non-uniformly refined again using C1 and then C2 symmetry for non-uniform and a local refinement. Once better aligned, particles were symmetry expanded and 3D classified again, then locally refined with C1 symmetry. Individual chains were then masked and subtracted to better resolve residues. The resulting maps were used to generate a model using Alphafold2 multimer, Rosetta v2021.16, Phenix v1.29.1-4487, Gaussian mixture model based atomic model refinement in EMAN2 v2.99.66, and Coot v0.9.8.92. The model of OGA is colored by domain, catalytic domain: dark blue, unstructured regions: gray, flexible helix: cyan; stalk: yellow. **c,** A representative fit of the model to the map, highlighting residues 113-124 in the catalytic domain, map is shown with gray mesh. **d,** OGA buried interfacial residues shown in three views (90° rotations) with the OGA model colored by domain: catalytic domain, dark blue; stalk, yellow highlighting the dimer interface. Interfacial residues in the dimer interface are shown as sticks with hydrogen bonding residues in orange. The buried surface area of the interface is 4203.3 Å^2, determined using PDBePISA [53].

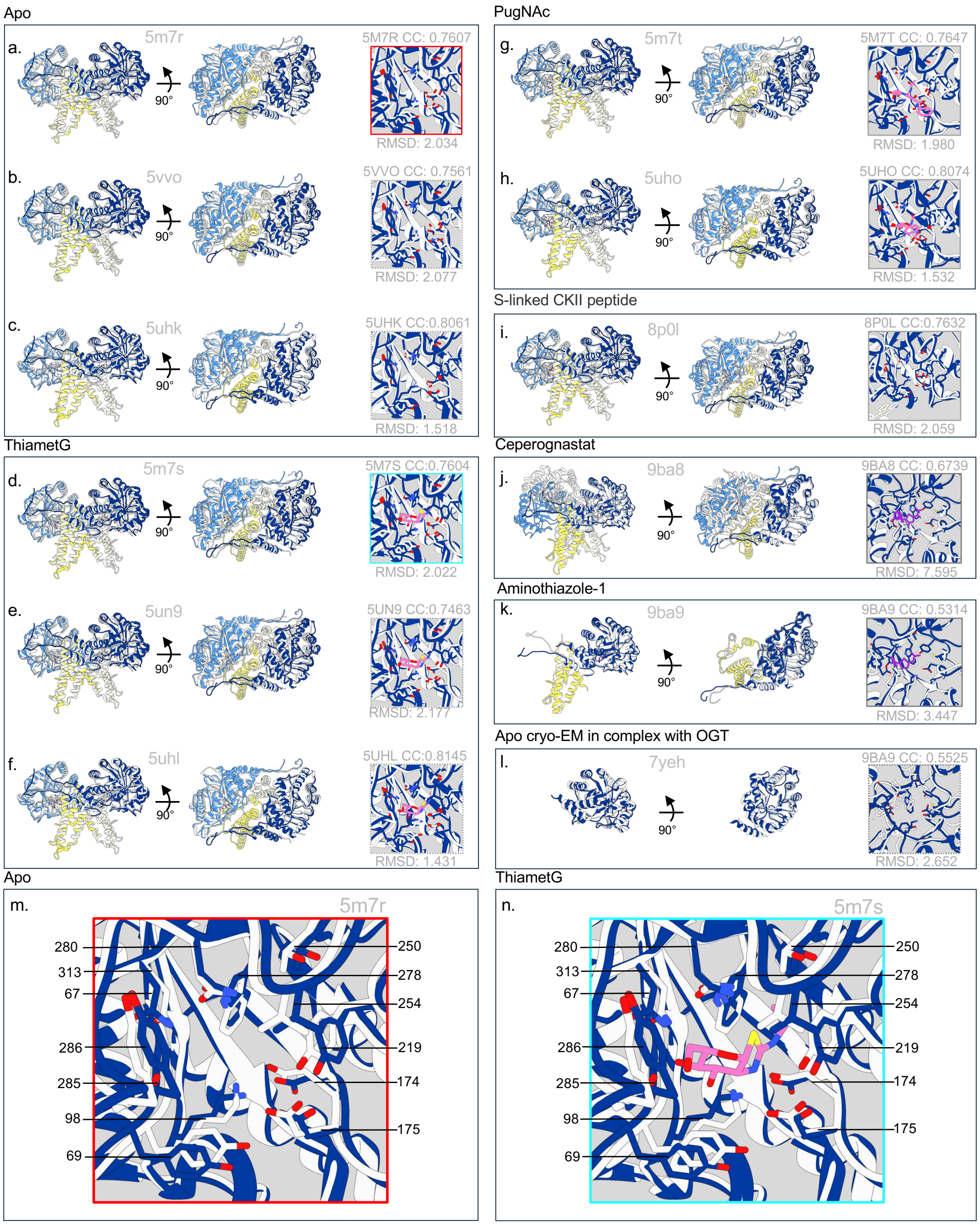

Supplementary Fig. 2: OGA model compared to crystal structures. a-k, cryo-EM OGA model colored by domain (catalytic domain, dark blue; stalk, yellow) overlaid with crystal structures (gray). Right, magnified view of the active site with or without substrate bound with cross-correlation and RMSD values between the models shown above and below respectively. **a-c**, Apo crystal structures, 5m7r, 5vvo, and 5uhk. **d-f**, ThiametG (pink) bound crystal structures 5m7s, 5un9, and 5uhl. **g-h**, PugNAc (pink) bound crystal structures 5m7t and 5uho. **i**, S-linked CDKII peptide bound crystal structure 8p0l. **j**, Ceperognastat (purple) bound crystal structure

9ba8. **k**, Aminothiazole-1 (purple) bound crystal structure 9ba9. **i**, OGA model colored as in (**a**) overlaid with the Apo cryo-EM of OGA in complex with OGT, 7yeh (gray). Right panel, magnified view of the active site, displaying residues involved in the ThiametG binding (dashed gray box) with cross-correlation and RMSD values between the models shown above and below. **m,n,** Zoom of OGA model colored blue overlaid with the Apo crystal structure 5m7r (**a**, red box) and the ThiametG bound crystal structure 5m7s (**d**, blue box) colored white highlighting the active site, with residues involved in ThiametG binding shown as sticks.

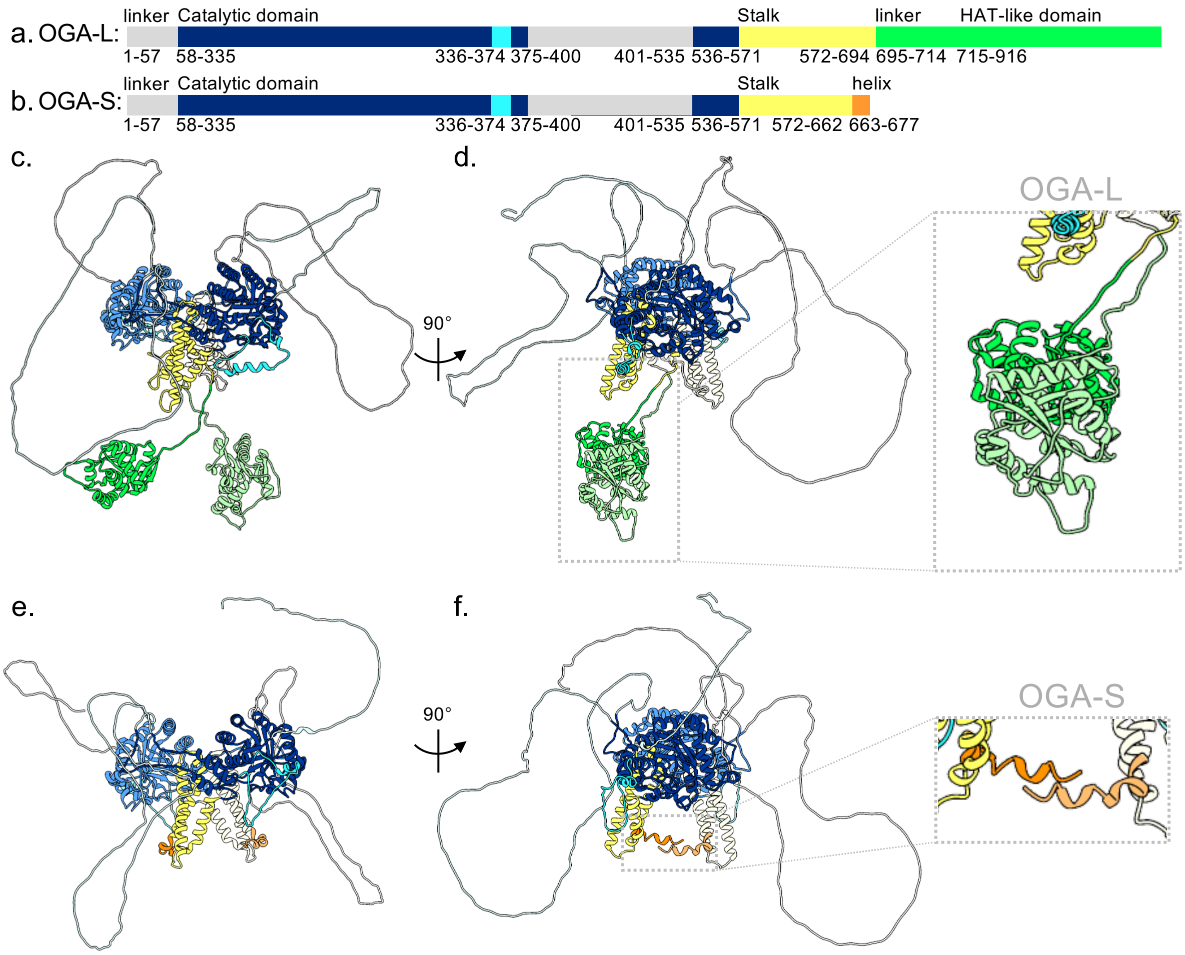

Supplementary Fig. 3: A comparison between OGA-L and OGA-S. a,b, Sequence diagram of OGA-L (**a**) and OGA-S colored by domain, highlighting flexible regions. OGA catalytic domain: dark blue, unstructured regions: gray, flexible helix: cyan; stalk: yellow; linker and HAT-like domain: green. **c**, OGA-L model with unstructured linkers added from Alphafold2 colored as in (**a**). **d,** A 90° rotation of the model, with a zoom (dashed gray box) highlighting the linker, and the HAT-like domain. **e,** An Alphafold2 model of OGA-S colored as in (**a**). **f,** A 90° rotation of the model, with a zoom (dashed gray box) highlighting the unique a- helical extension.

```
sp|OGA-S      MVQKESQATLEERESELSSNPAASAGASLEPPAAPAPGEDNPAGAGGAAVAGAAGGARRF  60
sp|OGA-3      MVQKESQATLEERESELSSNPAASAGASLEPPAAPAPGEDNPAGAGGAAVAGAAGGARRF  60
sp|OGA-L      MVQKESQATLEERESELSSNPAASAGASLEPPAAPAPGEDNPAGAGGAAVAGAAGGARRF  60
sp|OGA-4      MVQKESQATLEERESELSSNPAASAGASLEPPAAPAPGEDNPAGAGGAAVAGAAGGARRF  60
              ************************************************************

sp|OGA-S      LCGVVEGFYGRPWVMEQRKELFRRLQKWELNTYLYAPKDDYKHRMFWREMYSVEEAEQLM  120
sp|OGA-3      LCGVVEGFYGRPWVMEQRKELFRRLQKWELNTYLYAPKDDYKHRMFWREMYSVEEAEQLM  120
sp|OGA-L      LCGVVEGFYGRPWVMEQRKELFRRLQKWELNTYLYAPKDDYKHRMFWREMYSVEEAEQLM  120
sp|OGA-4      LCGVVEGFYGRPWVMEQRKELFRRLQKWELNTYLYAPKDDYKHRMFWREMYSVEEAEQLM  120
              ************************************************************

sp|OGA-S      TLISAAREYEIEFIYAISPGLDITFSNPKEVSTLKRKLDQVSQFGCRSFALLFDDIDHNM  180
sp|OGA-3      TLISAAREYEIEFIYAISPGLDITFSNPKEVSTLKRKLDQVSQFGCRSFALLFDDIDHNM  180
sp|OGA-L      TLISAAREYEIEFIYAISPGLDITFSNPKEVSTLKRKLDQVSQFGCRSFALLFDDIDHNM  180
sp|OGA-4      TLISAAREYEIEFIYAISPGLDITFSNPKEVSTLKRKLDQVSQFGCRSFALLFDDIDHNM  180
              ************************************************************

sp|OGA-S      CAADKEVFSSFAHAQVSITNEIYQYLGEPETFLFCPTEYCGTFCYPNVSQSPYLRTVGEK  240
sp|OGA-3      CAADKEVFSSFAHAQVSITNEIYQYLGEPETFLFCPTEYCGTFCYPNVSQSPYLRTVGEK  240
sp|OGA-L      CAADKEVFSSFAHAQVSITNEIYQYLGEPETFLFCPTEYCGTFCYPNVSQSPYLRTVGEK  240
sp|OGA-4      CAADKEVFSSFAHAQVSITNEIYQYLGEPETFLFCPTEYCGTFCYPNVSQSPYLRTVGEK  240
              ************************************************************

sp|OGA-S      LLPGIEVLWTGPKVVSKEIPVESIEEVSKIIKRAPVIWDNIHANDYDQKRLFLGPYKGRS  300
sp|OGA-3      LLPGIEVLWTGPKVVSKEIPVESIEEVSKIIKRAPVIWDNIHANDYDQKRLFLGPYKGRS  300
sp|OGA-L      LLPGIEVLWTGPKVVSKEIPVESIEEVSKIIKRAPVIWDNIHANDYDQKRLFLGPYKGRS  300
sp|OGA-4      LLPGIEVLWTGPKVVSKEIPVESIEEVSKIIKRAPVIWDNIHANDYDQKRLFLGPYKGRS  300
              ************************************************************

sp|OGA-S      TELIPRLKGVLTNPNCEFEANYVAIHTLATWYKSNMNGVRKDVVMTDSEDSTVSIQIKLE  360
sp|OGA-3      TELIPRLKGVLTNPNCEFEANYVAIHTLATWYKSNMNGVRKDVVM---------------  345
sp|OGA-L      TELIPRLKGVLTNPNCEFEANYVAIHTLATWYKSNMNGVRKDVVMTDSEDSTVSIQIKLE  360
sp|OGA-4      TELIPRLKGVLTNPNCEFEANYVAIHTLATWYKSNMNGVRKDVVM---------------  345
              *********************************************

sp|OGA-S      NEGSDEDIETDVLYSPQMALKLALTEWLQEFGVPHQYSSRQVAHSGAKASVVDGTPLVAA  420
sp|OGA-3      --------------------------------------SRQVAHSGAKASVVDGTPLVAA  367
sp|OGA-L      NEGSDEDIETDVLYSPQMALKLALTEWLQEFGVPHQYSSRQVAHSGAKASVVDGTPLVAA  420
sp|OGA-4      --------------------------------------SRQVAHSGAKASVVDGTPLVAA  367
                                                    **********************

sp|OGA-S      PSLNATTVVTTVYQEPIMSQGAALSGEPTTLTKEEEKKQPDEEPMDMVVEKQEETDHKND  480
sp|OGA-3      PSLNATTVVTTVYQEPIMSQGAALSGEPTTLTKEEEKKQPDEEPMDMVVEKQEETDHKND  427
sp|OGA-L      PSLNATTVVTTVYQEPIMSQGAALSGEPTTLTKEEEKKQPDEEPMDMVVEKQEETDHKND  480
sp|OGA-4      PSLNATTVVTTVYQEPIMSQGAALSGEPTTLTKEEEKKQPDEEPMDMVVEKQEETDHKND  427
              ************************************************************

sp|OGA-S      NQILSEIVEAKMAEELKPMDTDKESIAESKSPEMSMQEDCISDIAPMQTDEQTNKEQFVP  540
sp|OGA-3      NQILSEIVEAKMAEELKPMDTDKESIAESKSPEMSMQEDCISDIAPMQTDEQTNKEQFVP  487
sp|OGA-L      NQILSEIVEAKMAEELKPMDTDKESIAESKSPEMSMQEDCISDIAPMQTDEQTNKEQFVP  540
sp|OGA-4      NQILSEIVEAKMAEELKPMDTDKESIAESKSPEMSMQEDCISDIAPMQTDEQTNKEQFVP  487
              ************************************************************

sp|OGA-S      GPNEKPLYTAEPVTLEDLQLLADLFYLPYEHGPKGAQMLREFQWLRANSSVVSVNCKGKD  600
sp|OGA-3      GPNEKPLYTAEPVTLEDLQLLADLFYLPYEHGPKGAQMLREFQWLRANSSVVSVNCKGKD  547
sp|OGA-L      GPNEKPLYTAEPVTLEDLQLLADLFYLPYEHGPKGAQMLREFQWLRANSSVVSVNCKGKD  600
sp|OGA-4      GPNEKPLYTAEPVTLEDLQLLADLFYLPYEHGPKGAQMLREFQWLRANSSVVSVNCKGKD  547
              ************************************************************

sp|OGA-S      SEKIEEWRSRAAKFEEMCGLVMGMFTRLSNCANRTILYDMYSYVWDIKSIMSMVKSFVQW  660
sp|OGA-3      SEKIEEWRSRAAKFEEMCGLVMGMFTRLSNCANRTILYDMYSYVWDIKSIMSMVKSFVQW  607
sp|OGA-L      SEKIEEWRSRAAKFEEMCGLVMGMFTRLSNCANRTILYDMYSYVWDIKSIMSMVKSFVQW  660
sp|OGA-4      SEKIEEWRSRAAKFEEMCGLVMGMFTRLSNCANRTILYDMYSYVWDIKSIMSMVKSFVQW  607
              ************************************************************

sp|OGA-S      LGRCTRNNLFS--SNILSL-----------------------------------------  677
sp|OGA-3      LGCRSHSSAQFLIGDQEPWAFRGGLAGEF--------------QPPPLTPTSKVYTIRPY  653
sp|OGA-L      LGCRSHSSAQFLIGDQEPWAFRGGLAGEFQRLLPIDGANDLFFQPPPLTPTSKVYTIRPY  720
sp|OGA-4      LGCRSHSSAQFLIGDQEPWAFRGGLAGEFQRLLPIDGANDLFFQPPPLTPTSKVYTIRPY  667
              **  ::..     .:

sp|OGA-S      ------------------------------------------------------------  677
sp|OGA-3      FPKDEASVYKICREMYDDGVGLPFQSQPDLIGDKLVGGLLSLSLDYCFVLEDEDGICGYA  713
sp|OGA-L      FPKDEASVYKICREMYDDGVGLPFQSQPDLIGDKLVGGLLSLSLDYCFVLEDEDGICGYA  780
sp|OGA-4      FPKDEASVYKICREMYDDGVGLPFQSQPDLIGDKLVGGLLSLSLDYCFVLEDEDGICGYA  727

sp|OGA-S      ------------------------------------------------------------  677
sp|OGA-3      LGTVDVTPFIKKCKISWIPFMQEKYTKPNGDKELSEAEKIMLSFHEEQEVLPETFLANFP  773
sp|OGA-L      LGTVDVTPFIKKCKISWIPFMQEKYTKPNGDKELSEAEKIMLSFHEEQEVLPETFLANFP  840
sp|OGA-4      LGTVDVTPFIKKCKISWIPFMQEKYTKPNGDKELSEAEKIMLSFHEEQEVLPETFLANFP  787

sp|OGA-S      ------------------------------------------------------------  677
sp|OGA-3      SLIKMDIHKKVTDPSVAKSMMACLLSSLKANGSRGAFCEVRPDDKRILEFYSKLGCFEIA  833
sp|OGA-L      SLIKMDIHKKVTDPSVAKSMMACLLSSLKANGSRGAFCEVRPDDKRILEFYSKLGCFEIA  900
sp|OGA-4      SLIKMDIHKKVTDPSVAKSMMACLLSSLKANGSRGAFCEVRPDDKRILEFYSKLGCFEIA  847

sp|OGA-S      ---------------- 677
sp|OGA-3      KMEGFPKDVVILGRSL 849
sp|OGA-L      KMEGFPKDVVILGRSL 916
sp|OGA-4      KMEGFPKDVVILGRSL 863
```

Supplementary Fig. 4: Sequence alignment of OGA isoforms. The four isoforms of OGA are colored by domain. OGA catalytic domain: dark blue, unstructured regions: grey, flexible helix: cyan; stalk: yellow; trans helix present in OGA-L: purple; linker and HAT-like domain: green. The short isoform, OGA-S, lacks the HAT-like domain and includes a unique a-helix colored orange.

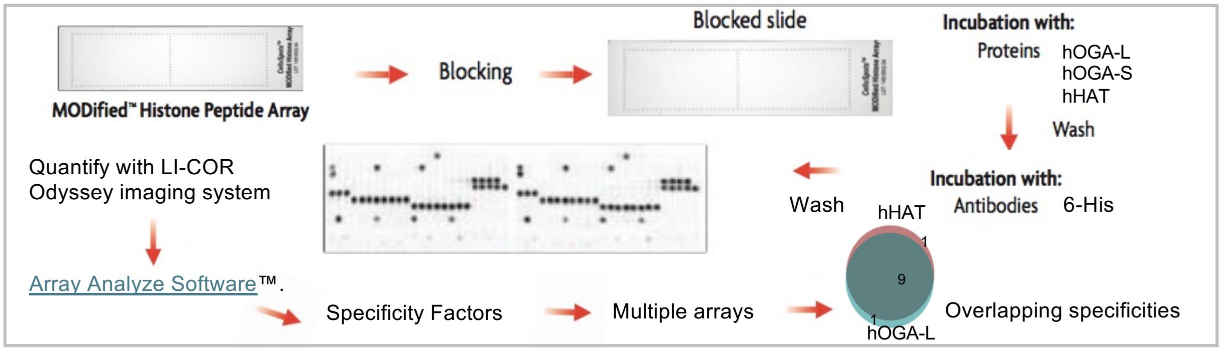

Supplementary Fig. 5. Workflow for Probing Modified™ Histone Arrays with OGA-L and OGA Subdomains. The OGA-L and each of the subdomains were expressed as fusion proteins with His-tag and C-term tag epitopes. Proteins were purified by affinity chromatography and after a blocking step were incubated with Histone arrays as described in Materials and Methods. After washing, arrays were probed with either anti-His Tag or C-term antibodies as described in materials and methods. After extensive washing, binding was quantified using a LI-COR Odyssey imaging system and analyzed using the Array Analyze Software to create a specificity index. Three of these duplicate arrays were analyzed and peptides showing specificity factors greater than 5 in all three probed duplicate arrays were judged to be specific binding interactions. Those interactions shared by the HAT and OGA-L domains were (in order of specificity factor): H3K36Me3>H3K36Me2>H3K36Ac>H3K36Me1>H3K36unmodifed>H4 1-19 K5,8,12,16Ac> H4K16Ac>H3R17Me2>H3R17Citr>H3K18A>H4R17Me2.

OGA-L (human)					
	Average Buried Surface Area (Å2)	Residues which H-bond	Residues which form salt bridges	Buried Interface Residues (A-chain)	Buried Interface Residues (B-chain)
**Catalytic dimer interface**	4203.3	C631 (A):Y101 (B), S629 (A):Y101 (B), E544 (A):N147 (B), N543 (A):A182 (B), N543 (A):A183 (B), Y641 (A):Q288 (B), D646 (A):K289 (B), E109 (A):H395 (B), D142 (A):Y397 (B), M105 (A):Y397 (B), E109 (A):S398 (B), T144(A):K545 (B), D142 (A):Y548 (B), E688 (A):H571 (B), L692 (A):R586 (B), D99 (A):R634 (B), D99 (A):Y638 (B), G70 (A):Y638 (B), P694 (A):W679 (B), Q690 (A):R682 (B), D287 (A):R682 (B), W679 (A):Q690 (B), Y101 (A):S629 (B), Y101 (A):C631 (B), R108 (A):Y397 (B), N147 (A):E544 (B), A182 (A):N543 (B), A183 (A):N543 (B), K289 (A):D646 (B), H395 (A):E109 (B), S398 (A):E109 (B), K545 (A):T144 (B), Y548 (A):D142 (B), Y548 (A):M105 (B), R586 (A):L692 (B), Y638 (A):G70 (B), W679 (A):P694 (B)	D675 (A):K253 (B), D646 (A):K289 (B), E109 (A):H395 (B), E688 (A):H571 (B), D287 (A):R682 (B), K253 (A):D675 (B), K289 (A):D646 (B), H395 (A):E109 (B)	Y69, G70, R71, P72, D99, D100, Y101, K102, M105, F106, W107, R108, E109, G140, L141, D142, I143, T144, N147, K149, E150, T153, D177, N179, M180, C181, A182, A183, T222, K253, D285, Y286, D287, Q288, K289, R290, H395, Q396, Y397, S398, E536, F538, N543, E544, K545, P546, L547, Y548, A550, D563, L564, F565, P568, Y569, E570, H571, G575, A576, M578, L579, F582, Q583, R586, F614, S629, N630, C631, A632, R634, Y638, D639, Y641, S642, Y643, W645, D646, I647, S649, I650, M651, M653, V654, F657, W660, Q670, F671, D675, Q676, E677, P678, W679, A680, F681, R682, G683, G684, L685, A686, E688, F689, Q690, R691, L692, L693, P694, I695, D696	Y69, G70, R71, P72, D99, D100, Y101, K102, M105, F106, R108, E109, G140, L141, D142, I143, T144, F145, N147, K149, E150, T153, D177, N179, M180, C181, A182, A183, T222, K253, D285, Y286, D287, Q288, K289, R290, C316, H395, Q396, Y397, S398, E536, Q537, F538, N543, E544, K545, P546, L547, Y548, A550, D563, L564, P568, Y569, E570, H571, G575, A576, M578, L579, F582, Q583, R586, F614, S629, N630, C631, A632, R634, Y638, D639, Y641, S642, Y643, W645, D646, I647, S649, I650, M651, M653, V654, F657, F671, D675, Q676, E677, W679, A680, F681, R682, G683, G684, L685, A686, G687, E688, F689, Q690, R691, L692, L693, P694, I695, D696

Supplementary Table 1: OGA-L dimer interface. Buried surface area and residues for each interface as identified by the server PDBePISA.[50]

Name	Product #	2˚ Antibody	Use in	Storage	manufacturer
H3	ab1791	Rabbit	M, H, C	Aliquoted -20˚	Abcam
H3 S28P	ab5169	Rabbit	H	Aliquoted -20˚	Abcam
H3 K36Ac	07-540	Rabbit	H	-20˚	Merck Millipore
H3 K36Me	ab9048	Rabbit	M,H	Aliquoted -20˚	Abcam
H3 K36Me2	ab9049	Rabbit	M, H, C	Aliquoted -20˚	Abcam
H3 K36Me3	ab9050	Rabbit	M, H, C	Aliquoted -20˚	Abcam
GFP	ab290	Rabbit	entire GFP	Aliquoted -20˚	Abcam
O-GlcNAc	MA1-076	Mouse		Aliquoted -20˚	Thermo Fisher
O-GlcNAc	MA1-072	Mouse		Aliquoted -20˚	Thermo Fisher
O-GlcNAc (RL2)	ab2739	Mouse		Aliquoted -20˚	Abcam
OGA C-term	SAB4200311	Rabbit	H	Aliquoted -20˚	Sigma Aldrich
OGT (H-300)	sc-32921	Rabbit	M, H	4˚	Santa Cruz
N-COAT (H-300)	sc-135093	Rabbit	M, H	4˚	Santa Cruz
OGA	sc-135093	Monoclonal Rabbit	M, H	Aliquoted -20˚	Santa Cruz
OGA	H00010724-MO2	Mouse	H	-80˚	Novus Biologicals
Anti-6His	His.H8	mouse	H	-80˚	Thermo Fisher
Anti-Myc	9E10	Mouse	H	-80˚	Thermo-Fisher
Anti-c-term	3D5	mouse	H	-80˚	Thermo Fisher

Supplementary Table 2: Antibody table.

Data collection and processing	OGA-L Catalytic Dimer (EMDB:EMD-49293, PDB ID: 9EN2)	OGA-L Catalytic Dimer A-Chain (EMDB:EMD-49294, PDB ID:	OGA-L Catalytic Dimer B-Chain (EMDB:EMD-49295, PDB ID:	OGA-L Catalytic Dimer with extra A-chain density (EMDBEMD-49297)	OGA-L Dimer (EMDB:EMD-49296)
Microscope	Titan Krios	Titan Krios	Titan Krios	Titan Krios	Titan Krios
Magnification	105,000x	105,000x	105,000x	105,000x	105,000x
Voltage (kV)	300	300	300	300	300
Electron exposure (e-/Å ^{2})	67	67	67	67	67
Defocus range (-μm)	0.3 to 2.4	0.3 to 2.4	0.3 to 2.4	0.3 to 2.4	0.3 to 2.4
spherical abberation (mm)	2.7	2.7	2.7	2.7	2.7
automation software	SerialEM	SerialEM	SerialEM	SerialEM	SerialEM
Data processing software	CryoSPARC v4.6.0	CryoSPARC v4.6.0	CryoSPARC v4.6.0	CryoSPARC v4.6.0	CryoSPARC v4.6.0
Pixel size (Å)	1.2114	1.2114	1.2114	1.2114	1.2045
Symmetry imposed	C1	C1	C1	C1	C1
total number of micrographs (no.)	1,737	1,737	1,737	1,737	1,737
Final particles (no.)	65,831	65,831	65,831	62,230	8,527
Box Size (pixels)	280	280	280	280	440
Map resolution (Å)	3.630	2.981	3.048	3.860	10.070
FSC threshold	0.143	0.143	0.143	0.143	0.143
**Refinement**					
Initial model	Alphafold2 Multimer	Alphafold2 Multimer	Alphafold2 Multimer		
Refinement Packages	Phenix v1.29.1-4487 Coot v0.9.8.92 EMAN2 v2.99.66	Phenix v1.29.1-4487 Coot v0.9.8.92 EMAN2 v2.99.66	Phenix v1.29.1-4487 Coot v0.9.8.92 EMAN2 v2.99.66		
Chains (no.)	2	1	1		
Model resolution (Å)	3.70	3.90	3.90		
FSC threshold	0.143	0.143	0.143		
Map CC	0.82	0.61	0.60		
Model composition					
Non-hydrogen atoms	7,652	3,826	3,826		
Protein residues	936	468	468		
Ligands	0	0	0		
B-factor					
Protein (Å ^{2}, mean)	89.56	89.48	89.63		
Validation					
MolProbity Score	1.28	1.19	1.30		
Clashscore	5.29	4.1	5.55		
Ramachandran plot					
Favored (%)	98.38	98.05	98.70		
Allowed (%)	1.62	1.95	1.30		
Disallowed (%)	0.00	0.00	0.00		
Rotamers outliers (%)	0.12	0.24	0.00		
Cβ outliers (%)	0.00	0.00	0.00		
R.M.S. deviations					
Bond Length (Å)	0.003 (0)	0.003 (0)	0.004 (0)		
Bond Length (°)	0.503 (1)	0.511 (1)	0.908 (4)		
Peptide plane (%)					
Cis proline/general (%)	4.0/0.0	4.0/0.0	4.0/0.0		
twisted proline/general (%)	0.0/0.0	0.0/0.0	0.0/0.0		
CaBLAM outliers (%)	0.99	0.88	1.10		

Supplementary Table 3: Cryo-EM data collection, refinement, and validation statistics.

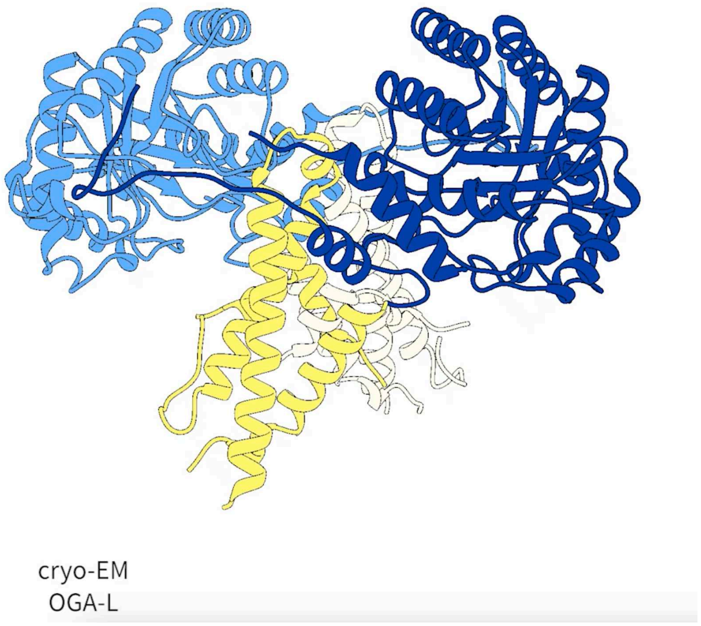

Supplementary Video 1: Morph between the cryo-EM model of the Apo OGA-L and the 5m7r crystal structure. Video shows a side and top view of the morph between the two models. OGA catalytic domain is blue, and the stalk is yellow.

Supplementary Files

This is a list of supplementary files associated with this preprint. Click to download.

- Video1.mp4
